# Clinical management of children with fever: a cross-sectional study of quality of care in rural Zambia

**DOI:** 10.2471/BLT.16.170092

**Published:** 2017-04-05

**Authors:** Karsten Lunze, Godfrey Biemba, J Joseph Lawrence, William B MacLeod, Kojo Yeboah-Antwi, Kebby Musokotwane, Toyin Ajayi, Simon Mutembo, Chilunga Puta, Duncan Earle, Rick Steketee, Davidson H Hamer

**Affiliations:** a Department of Medicine, Boston University Medical School, 801 Massachusetts Ave, Boston MA 02119, United States of America (USA).; b Zambian Centre for Applied Health Research and Development, Lusaka, Zambia.; c Global Health Corps Fellowship, Zambian Centre for Applied Health Research and Development, Lusaka, Zambia.; d Center for Global Health and Development, Boston University School of Public Health, Boston, USA.; e Ministry of Health, Lusaka, Zambia.; f Commonwealth Care Alliance, Boston, USA.; g Southern Provincial Medical Office, Ministry of Health, Choma, Zambia.; h PATH, Lusaka, Zambia.; i Malaria Control and Elimination Partnership in Africa Program, PATH, Seattle, USA.

## Abstract

**Objective:**

To evaluate current practices and standards of evaluation and treatment of childhood febrile illness in Southern Province, Zambia.

**Methods:**

From November to December 2013, we conducted a cross-sectional survey of facilities and health workers and we observed the health workers’ interactions with febrile children and their caregivers. The facility survey recorded level of staffing, health services provided by the facility, availability and adequacy of medical equipment, availability of basic drugs and supplies and availability of treatment charts and guidelines. The health worker survey assessed respondents’ training, length of service, access to national guidelines and job aids for managing illnesses, and their practice and knowledge on management of neonatal and child illnesses. We also conducted exit interviews with caregivers to collect information on demographic characteristics, chief complaints, counselling and drug dispensing practices.

**Findings:**

This study included 24 health facilities, 53 health workers and 161 children presenting with fever. Facilities were insufficiently staffed, stocked and equipped to adequately manage childhood fever. Children most commonly presented with upper respiratory tract infections (46%; 69), diarrhoea (31%; 27) and malaria (10%; 16). Health workers insufficiently evaluated children for danger signs, and less than half (47%; 9/19) of children with pneumonia received appropriate antibiotic treatment. Only 57% (92/161) were tested for malaria using either rapid diagnostic tests or microscopy.

**Conclusion:**

Various health system challenges resulted in a substantial proportion of children receiving insufficient management and treatment of febrile illness. Interventions are needed including strengthening the availability of commodities and improving diagnosis and treatment of febrile illness.

## Résumé

**Objectif:**

Évaluer les pratiques et les normes actuelles d'évaluation et de traitement des maladies fébriles infantiles dans la Province méridionale de la Zambie.

**Méthodes:**

De novembre à décembre 2013, nous avons mené une enquête transversale auprès d‘établissements et de professionnels de santé et nous avons observé les interactions entre les professionnels de santé, les enfants fiévreux et leurs proches. L'enquête réalisée dans les établissements s'est intéressée à la dotation en personnel, aux services sanitaires dispensés par l'établissement, à la disponibilité et à la convenance des équipements médicaux, à la disponibilité de médicaments et de fournitures élémentaires et à la présence de directives et de feuilles d'observation. L'enquête réalisée auprès des professionnels de santé a analysé la formation des répondants, leur nombre d'années de service, leur accès aux directives nationales et aux aide-mémoires pour la prise en charge des maladies, ainsi que leur pratique et leurs connaissances en matière de prise en charge des maladies infantiles et néonatales. Nous avons également interrogé les proches à leur sortie afin de recueillir des informations sur les caractéristiques démographiques, les principales plaintes, les conseils et les pratiques d'administration des médicaments.

**Résultats:**

Cette étude a examiné 24 établissements de santé, 53 professionnels de santé et 161 enfants atteints de fièvre. Les établissements manquaient de personnel, de produits en stock et d'équipements pour prendre en charge correctement les cas de fièvre infantile. Les enfants étaient le plus souvent atteints d'infections des voies respiratoires supérieures (46%; 69), de diarrhée (31%; 27) et de paludisme (10%; 16). Les professionnels de santé n'évaluaient pas suffisamment les signes de danger chez les enfants, et moins de la moitié (47%; 9/19) des enfants atteints de pneumonie ont reçu un traitement antibiotique adapté. Seuls 57% (92/161) ont fait l'objet d'un dépistage du paludisme, au moyen de tests de diagnostic rapide ou par microscopie.

**Conclusion:**

À cause des différents problèmes du système sanitaire, une part importante d'enfants ne bénéficie pas d'une prise en charge et d'un traitement suffisants des maladies fébriles. Des interventions sont nécessaires, notamment pour augmenter la disponibilité des produits et améliorer le diagnostic et le traitement des maladies fébriles.

## Resumen

**Objetivo:**

Evaluar las prácticas y los estándares de evaluación y tratamiento actuales de las enfermedades febriles en los niños en la Provincia del Sur, Zambia.

**Métodos:**

De noviembre a diciembre de 2013, se realizó una encuesta transversal de centros y trabajadores sanitarios y se observaron las interacciones de los trabajadores sanitarios con niños con fiebre y sus cuidadores. La encuesta de los centros registró el nivel de personal y servicios de salud ofrecidos en el centro, la disponibilidad e idoneidad de los equipos médicos, la disponibilidad de medicamentos y suministros básicos y la disponibilidad de cronogramas y directrices de tratamiento. La encuesta a los trabajadores sanitarios evaluó la formación de los encuestados, la duración del servicio, el acceso a directrices nacionales y ayudas laborales para la gestión de enfermedades y su práctica y conocimiento acerca de la gestión de las enfermedades neonatales e infantiles. También se realizaron entrevistas de salida con cuidadores para recopilar información acerca de las características demográficas, los motivos de consulta, asesoramiento y prácticas de distribución de medicamentos.

**Resultados:**

Este estudio abarcó 24 centros sanitarios, 53 trabajadores sanitarios y 161 niños con fiebre. Los centros no contaban con personal, suministros y equipos suficientes para gestionar de forma adecuada la fiebre en niños. Las afecciones más comunes presentadas por los niños eran infecciones del tracto respiratorio superior (46%; 69), diarrea (31%; 27) y malaria (10%; 16). Los trabajadores sanitarios evaluaron a los niños de forma insuficiente en busca de signos de peligro, y menos de la mitad (47%; 9/19) de los niños con neumonía recibieron el tratamiento antibiótico adecuado. Solo el 57% (92/161) fue sometido a pruebas de malaria utilizando pruebas de diagnóstico rápido o microscópicas.

**Conclusión:**

Varios desafíos del sistema sanitario llevan a un gran porcentaje de niños que reciben una gestión y tratamiento insuficientes de las enfermedades febriles. Es necesario intervenir para fortalecer la disponibilidad de productos básicos y mejorar el diagnóstico y el tratamiento de las enfermedades febriles.

## ملخص

**الغرض:**

تقييم الممارسات الحالية ومعايير التقييم ومعالجة مرض الحمى لدى الأطفال في المنطقة الجنوبية في زامبيا.

**الطريقة:**

في الفترة ما بين نوفمبر/تشرين الثاني إلى ديسمبر/كانون الأول 2013، قمنا بإجراء استقصاء مقطعي للمرافق الصحية والعاملين بمجال الصحة ولاحظنا تفاعلات العاملين بمجال الصحة مع الحمى لدى الأطفال ومقدمي الرعاية لهم. وكان المسح الذي تم على مرافق الرعاية الصحية قد سجّل مستوى التوظيف، والخدمات الصحية المقدمة من قبل المِرفق، وتوافر المعدات الطبية ومدى كفاءتها، ومدى توافر المستلزمات والأدوية الأساسية، وتوافر الجداول التوضيحية وإرشادات العلاج. كما عمل المسح الذي تم على العاملين في مجال الصحة على تقييم التدريب الذي خضع له المشاركون، ومدة الخدمة، وإمكانية الاطلاع على الإرشادات القومية والأدوات المساعدة على العمل للسيطرة على المرض، والإجراءات المتبعة من جانبهم، ومعرفتهم بإدارة أمراض الأطفال وحديثي الولادة. كما قمنا أيضًا بإجراء مقابلات نهائية مع مقدمي الرعاية لجمع المعلومات المتعلقة بالخصائص الديموغرافية، والشكاوى الأساسية، والاستشارة والإجراءات المتبعة لصرف الدواء.

**النتائج:**

تضمنت هذه الدراسة 24 مرفقًا صحيًا، و53 عاملاً في مجال الصحة، و161 طفلاً معرضًا للحمى. وُجد أن المرافق لم يتوفر بها ما يكفي من الموظفين، وغير مؤهلة ولا مجهزة لإدارة الحمى لدى الأطفال بشكلٍ ملائم. أغلب الأطفال كانوا مصابين بالتهاب الجهاز التنفسي العلوي (‏46‏%، 69)، والإسهال (31‏%، 27)، والملاريا (10‏%، 16). ولم يقم العاملون في مجال الصحة بتقييم الأطفال للكشف عن الأعراض الخطيرة بشكلٍ كافِ، وكان أقل من نصف الأطفال (47‏%، 9/19) المصابين بالالتهاب الرئوي قد تلقوا علاجًا مناسبًا بالمضادات الحيوية. ولم يتم فحص إلا 57‏% (92/161) للكشف عن الإصابة بالملاريا باستخدام إما الاختبارات التشخيصية السريعة أو الاختبارات المجهرية.

**الاستنتاج:**

تؤدي التحديات المختلفة التي يتعرض لها النظام الصحي إلى وجود نسبة كبيرة من الأطفال ممن يتلقون علاجًا وإدارة غير كافية لمرض الحمى. ويلزم إجراء التدخلات بما في ذلك ما كان منها لتعزيز توافر التجهيزات وتحسين تشخيص مرض الحمى وعلاجه.

## 摘要

**目的:**

旨在评估赞比亚南部省当前的小儿热病评估和治疗实践和标准。

**方法:**

我们从 2013 年 11 月到 12 月对医疗机构和医务人员进行了横断面调查，并且观察了医务人员与发热儿童及其看护人之间的互动。 医疗机构调查记录了人员配备水平、医疗机构提供的卫生服务、医疗设备的可用性和充足性、基本药物和用品的可用性以及治疗计划和指南的可用性。 医务人员调查评估了受访者的培训、服务时长、获取全国疾病管理指南和工作支持的机会，以及他们在新生儿和小儿疾病管理方面的实践和知识。 我们也对看护人进行了离职访问，以收集有关人口统计特征、主诉、咨询和配药实践的信息。

**结果:**

本次调查涵盖 24 家医疗机构，53 名医务人员和 161 名出现发热症状的儿童。 这些医疗机构存在人员、医疗用品和设备配备不足的情况，无法对儿童发热进行良好的管理。 儿童最常见的症状是上呼吸道感染 (46%; 69)、腹泻 (31%; 27) 和疟疾 (10%; 16)。 医务人员无法充分评估儿童的危险症状，只有不到一半 (47%; 9/19) 的肺炎儿童接受了适当的抗生素治疗。 只有 57% (92/161) 的儿童通过快速诊断测试或显微镜检查接受了疟疾检测。

**结论:**

卫生系统面临的多种挑战导致大部分儿童无法接受足够的热病管理和治疗。 需要在提高商品可用性、改善热病诊断和治疗等方面加强干预。

## Резюме

**Цель:**

Оценить текущую практику и стандарты оценки и лечения детских заболеваний, сопровождающихся лихорадкой, в Южной провинции, Замбия.

**Методы:**

С ноября по декабрь 2013 года мы провели одномоментное обследование медицинских учреждений и медицинских работников, наблюдая за тем, как медицинские работники взаимодействуют с детьми, страдающими лихорадкой, и с лицами, осуществляющими за ними уход. В В ходе обследования медицинских учреждений фиксировался уровень укомплектованности кадрами и медицинских услуг, предоставляемых этими учреждениями, наличие и соответствие медицинского оборудования, наличие основных лекарств и расходных материалов, а также наличие руководств по лечению. В ходе обследования медицинских работников была проведена оценка их подготовки, стажа работы, доступа к национальным руководствам и инструкциям по лечению заболеваний, а также их практики и знаний относительно лечения болезней новорожденных и детей. Мы также провели беседы с лицами, ухаживающими за больными, для сбора информации о демографических характеристиках, основных жалобах, консультировании и практике выдачи лекарств.

**Результаты:**

Это исследование включало 24 медицинских учреждения, 53 медицинских работника и 161 ребенка с лихорадкой. Учреждения были недостаточно укомплектованы персоналом и имели недостаточное оснащение и снабжение для осуществления надлежащего лечения лихорадки у детей. У детей чаще всего встречались инфекции верхних дыхательных путей (46%, 69), диарея (31%, 27) и малярия (10%, 16). Медицинские работники проводили недостаточное обследование детей на наличие опасных симптомов, и менее половины детей с пневмонией (47%, 9 из 19) получали соответствующее лечение антибиотиками. Только 57% (92 из 161) были протестированы на малярию с использованием либо экспресс-диагностических тестов, либо микроскопии.

**Вывод:**

Различные проблемы системы здравоохранения приводят к тому, что значительная часть детей получает недостаточное лечение заболеваний, сопровождающихся лихорадкой. Необходимо принять меры, включая повышение доступности лекарств и оборудования, а также улучшение диагностики и лечения заболеваний, сопровождающихся лихорадкой.

## Introduction

In Africa, historically, fever in children younger than 5 years has been considered to be due to malaria and has been treated immediately with antimalarial drugs without laboratory confirmation to avoid treatment delays.^1^ The recent reduction in malaria-related morbidity and mortality^2^ has prompted a change in clinical management of children presenting with fever.^3^^–^^5^ In 2010, the World Health Organization (WHO) updated its guidelines from presumptive antimalarial treatment of children with fever to recommend diagnostic testing for malaria, by either microscopy or malaria rapid diagnostic test, to ensure rational use of antimalarials.^6^

Reflecting global trends, Zambia has recently made progress in reducing its malaria-associated disease burden by about 7% annually from 2000 to 2010,^7^^,^^8^ resulting in over 50% reduction in malaria deaths.^9^

Health-care services in Zambia are delivered through a five-level hospital system, which includes three tertiary hospitals, 21 provincial or general hospitals and 85 district hospitals, employing specialist staff. These hospitals receive referrals from urban and rural health centres and community-based health posts at the lowest levels of the health-care system throughout the country. Health care in Zambia faces chronic challenges with human resources and inadequate supplies of drugs, equipment and medical supplies. Starting in 1996, the Zambian Ministry of Health adopted an integrated management of childhood illness (IMCI) approach to managing the causes of childhood febrile illness, including malaria, viral or bacterial infections.^10^ The health ministry has made several adaptations to the generic IMCI guidelines issued by WHO and the United Nations Children’s Fund (UNICEF), to include guidelines for human immunodeficiency virus (HIV), diarrhoea management, use of malaria rapid diagnostic test for classifying fever and newborn care. In the country, the principles of the IMCI guidelines are incorporated into pre-service curricula and training programmes and the approach is implemented in all districts. However, less than 10% of the districts have the minimum level of 80% health workers trained in IMCI as required by the ministry of health.^11^ As a result, health workers in Zambia face the challenge of managing febrile illness when malaria test results are negative.^12^

With this study, we aimed to evaluate current practices and standards of care for childhood febrile illness at different levels of the health-care system, in Southern Province, Zambia.

## Methods

### Study site

In 2013, Southern Province had an estimated population of about 1.6 million.^13^ It is one of 10 provinces in Zambia and is divided into 13 districts.^13^ Malaria transmission in the province ranges from hypoendemic (less than 10% of children aged 2–9 years are parasitemic) to holoendemic (more than 75% of children aged 2–9 years are parasitemic). In recent years, Southern Province has been the focus of intensive malaria control activities using reactive case detection^14^ and mass malaria test and treat strategies, with the aim of creating malaria-free areas within the province.^15^ In 2012, in Southern Province, 44% of children younger than 5 years slept under a treated bednet and 22% of all households reported indoor residual spraying in the previous 12 months.^16^

### Study design

We conducted two types of cross-sectional surveys; one for facilities and one for health workers. Fundamental aspects of the survey design and execution were described previously.^17^ The facility survey was used to record level of staffing, health services provided by the facility, availability and adequacy of medical equipment, availability of basic drugs and supplies and availability of treatment charts and guidelines. We also reviewed outpatient, inpatient and laboratory or malaria rapid diagnostic test registers to document the number of children consulted at the facility, proportions with febrile illness and proportions of confirmed cases of malaria who presented with a febrile illness in the last month. The health worker survey was used to assess respondent’s training, length of service, access to national guidelines, use of wall charts, algorithms/decision charts or other job aids for managing illnesses, and their practice and knowledge on management of neonatal and child illnesses. In addition to the surveys, we observed the health workers’ interactions with febrile children and their caregivers to assess history taking and examination, investigations performed or ordered to aid diagnosis and actions taken, including treatments and counselling. We recorded the observation by using a structured data collection tool that was designed to assess all relevant steps in taking a history and examining a child, as described in the national IMCI guidelines. We also conducted brief structured exit interviews with caregivers (lasting less than five minutes) to collect de-identified information on demographic characteristics, chief complaint, counselling and drug dispensing practices. During the exit interview, we also reviewed the child’s clinic card and documented the care received including diagnosis made, investigations ordered and results and treatment given. These tools were adapted from previously used tools,^17^ and were pilot tested in a health centre outside Southern Province. All survey instruments are available from the authors.

The Institutional Review Boards of Boston University Medical Center, ERES Converge, (a local institutional review board in Lusaka, Zambia), the Zambian Ministry of Health and the Southern Provincial Medical Office approved the study.

### Sampling

Our sampling frame included three districts in Southern Province with differing levels of malaria transmission: Kazungula (low), Kalomo (moderate) and Siavonga (high). In the study area, none of the three districts had a level 3 (tertiary) hospital. Therefore, we sampled from level 2 (district hospitals) and level 1 (health centres), which in Zambia serve the highest volume of febrile children.

All health workers attending to febrile children and caregivers 18 years or older were eligible to be included in the study. Health workers caring for febrile children with complications requiring referral, and their caregivers; as well as caregivers unable or unwilling to provide written informed consent were excluded from the study.

### Data collection

We collected the data from November to December 2013, a time of year in Zambia when malaria incidence starts to increase. We obtained written informed consent from all study participants in English or Chitonga, the local language in Southern Province.

### Study outcomes and analysis

**Fig. 1:**
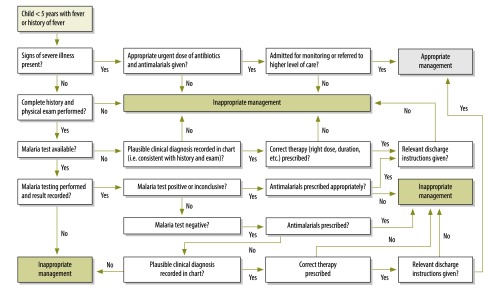
Flowchart showing the development of the study’s primary outcome, appropriate fever management

Note: Fever management best practice evaluation algorithm derived from the *Integrated technical guidelines for frontline health workers*^18^ and the *Guidelines for diagnosis and treatment of malaria in Zambia*.^19^

The primary outcome was the proportion of children with febrile illness (temperature above 37.5 °C or history of subjective fever in the previous 48 hours) managed according to the guidelines issued by the health ministry.^10^The guidelines include identification of seven signs and symptoms of febrile illness: four danger signs for childhood illnesses in outpatient settings (poor oral intake, vomiting everything, convulsions and decreased level of consciousness); ensuring appropriate combined treatment of all major illnesses; strengthening the counselling of caregivers; and referring severely ill children.^10^ For our outcome, we defined appropriate management based on Zambia’s *Integrated technical guidelines for frontline health workers*^18^ and the *Guidelines for the diagnosis and treatment of malaria in Zambia*^19^ (Fig. 1). While both of these sets of guidelines are based on the IMCI guidelines, there is greater depth in the malaria guidelines.^19^

Secondary outcomes included: (i) proportion of children presenting with positive blood smear or rapid diagnostic test result and with medical conditions most commonly associated with non-malaria febrile illness; (ii) proportion of health facilities with numbers and cadres of staff as stated in ministry of health guidelines and established targets needed to manage childhood febrile illness; (iii) proportion of health facilities with essential equipment, supplies and drugs to manage childhood febrile illness; (iv) proportion of health workers in-service and pre-service training in the management of childhood febrile illness, such as IMCI and malaria.

We calculated frequencies for categorical variables and means and standard errors for continuous variables.

## Results

We surveyed 24 health facilities (comprising four hospital-affiliated urban health centres and 20 rural health centres) and 53 health workers (12 physicians or clinical officers, 26 nurses, eight environmental health technicians and seven other staff) who provided care for febrile children. Table 1 summarizes the characteristics of the surveyed facilities. We also observed the assessment and management of 161 febrile children, with mean age of 24.1 months. Almost half (49.7%; 80 children) were male.

**Table 1:** Characteristics of 24 health facilities surveyed, by facility type, Southern Province, Zambia, 2013

Characteristic	No. (%)		
	HAHC (*n* = 4)	RHC (*n* = 20)	All facilities (*n* = 24)
**Type of staff**			
Physician, clinical officer or nurse	4 (100.0)	19 (95.0)	23 (95.8)
Physician	3 (75 .0)	0 (0.0)	3 (12.5)
Clinical officer or medical licentiate	4 (100.0)	5 (25.0)	9 (37.5)
Midwife	0 (0.0)	9 (45.0)	9 (37.5)
Nurse or registered nurse	2 (50.0)	18 (90.0)	20 (83.3)
Other (EHT, CHA or CHW)	1 (25.0)	17 (85.0)	18 (75.0)
**Availability of diagnostics**			
Functional microscope	3 (75.0)	6 (30.0)	9 (37.5)
Malaria RDTs	4 (100.0)	18 (90.0)	22 (91.7)
Functional pulse oximeter	0 (0.0)	0 (0.0)	0 (0.0)
Functional X-ray machine	3 (75.0)	1 (5.0)	4 (16.7)
Respiratory rate counter or watch with indicator for seconds	1 (25.0)	5 (25.0)	6 (25.0)
RDT wall chart displayed	0 (0.0)	5 (25.0)	5 (20.8)
IMCI wall chart displayed	0 (0.0)	3 (15.0)	3 (12.5)
AL dosing wall chart displayed	0 (0.0)	6 (30.0)	6 (25.0)
At least one wall chart displayed (malaria RDT, AL dosing or IMCI)	0 (0.0)	10 (50.0)	10 (41.7)
All three wall charts displayed (malaria RDT, AL dosing and IMCI)	0 (0.0)	1 (5.0)	1 (4.2)
**Availability of drugs**			
AL in stock on day of survey	4 (100.0)	20 (100.0)	24 (100.0)
AL stock-outs in previous 12 months	0 (0.0)	2 (10.0)	2 (8.3)
Amoxicillin in stock on day of survey	3 (75.0)	19 (95.0)	22 (91.7)
Amoxicillin stock-outs in previous 12 months	1 (25.0)	8 (40.0)	9 (37.5)

AL: artemether-lumefantrine; CHA: community health assistant; CHW: community health worker; EHT: environmental health technician; HAHC: hospital affiliated health centre; ICMI: integrated management of childhood illness; RHC: rural health centre; RDT: rapid diagnostic test.

### Diagnostic and treatment capacity

While all 24 surveyed facilities had diagnostic capacity for malaria, 83.3% (20) lacked other diagnostic equipment such as a functional X-ray machine and 75% (18) lacked an instrument for counting respiratory rates. Twenty-three facilities (95.8%) lacked visual job aids, such as wall charts and diagnostic and treatment guidance. While all health facilities had artemether-lumefantrine for treatment of non-severe malaria, only 92% (22) stocked antibiotics for pneumonia, but drug stock-outs were common.

### Training

Table 2 presents the characteristics of the surveyed health workers, training they received and the results of their skill assessment. Over half (50.9%; 27 health workers) had recent formal training in either malaria case management, IMCI, or integrated community case management. Less than one third (32.1%; 17 health workers) had access to IMCI guidelines, while 24.5% (13) had access to both integrated community case management as well as malaria treatment guidelines.

**Table 2:** Health-care workers’ characteristics, training and skill assessment performance in 24 health facilities, Southern Province, Zambia, 2013

Characteristic	Physician, clinical officer (*n* = 12)	Nurse (*n* = 26)	EHT (*n* = 8)	Other^a^ (*n* = 7)	All health workers (*n* = 53)
**Mean age in years (SE)**	35.8 (2.5)	32.5 (1.4)	32.4 (2.4)	38.0 (3.8)	33.9 (1.1)
**Male, no. (%)**	9 (75.0)	11 (42.3)	6 (75.0)	4 (57.1)	30 (56.6)
**Mean years of professional experience (SE)**	5.7 (2.2)	6.1 (1.3)	8.3 (2.5)	8.0 (2.2)	6.6 (0.9)
**Mean years of working at this facility (SE)**	5.1 (1.7)	3.7 (0.8)	5.8 (2.5)	7.9 (2.4)	4.9 (0.7)
**Working at facility at least 3 years, no. (%)**	6 (50.0)	11 (42.3)	6 (75.0)	4 (57.1)	27 (50.9)
**Training received^b^**					
Malaria case management,^c^ no. (%)	4 (33.3)	3 (11.5)	3 (37.5)	1 (14.3)	11 (20.8)
IMCI, no (%)	7 (58.3)	7 (26.9)	1 (12.5)	1 (14.3)	16 (30.2)
iCCM, no. (%)	3 (25.0)	1 (3.8)	1 (12.5)	0 (0.0)	5 (9.4)
At least one mCCM, IMCI or iCCM, no. (%)	10 (83.3)	11 (42.3)	5 (62.5)	1 (14.3)	27 (50.9)
**Access to guidelines in facility**					
Integrated management of childhood illness guidelines, no. (%)	3 (25.0)	12 (46.2)	2 (25.0)	0 (0.0)	17 (32.1)
Integrated community case management guidelines, no. (%)	4 (33.3)	8 (30.8)	1 (12.5)	0 (0.0)	13 (24.5)
Malaria treatment guidelines, no. (%)	2 (16.7)	9 (34.6)	1 (12.5)	1 (14.3)	13 (24.5)
**Skills assessment performance**					
IMCI danger signs and symptoms^d^					
At least three of seven items, no. (%)	11 (91.7)	26 (100.0)	7 (87.5)	6 (85.7)	50 (94.3)
All seven items, no. (%)	3 (25.0)	4 (15.4)	1 (12.5)	0 (0.0)	8 (15.1)
Correct disease classification^e^					
At least three of seven items, no. (%)	12 (100.0)	23 (88.5)	7 (87.5)	4 (57.1)	46 (86.8)
All seven items, no. (%)	0 (0.0)	0 (0.0)	0.(0.0)	0 (0.0)	0 (0.0)
Correct treatment^f^					
At least three of seven items, no. (%)	2 (16.7)	7 (26.9)	1 (12.5)	0 (0.0)	10 (18.9)
All seven items, no. (%)	0 (0.0)	0 (0.0)	0. (0.0)	0 (0.0)	0 (0.0)

EHT: environmental health technician; iCCM: integrated community case management; IMCI : integrated management of childhood illness; mCCM : malaria community case management; SE: standard error.

^a^ Includes 4 cleaning and maintenance staff, 1 community health assistant, 1 community health worker and 1 data clerk.

^b^ Proportion received in-service training (previous 3 years).

^c^ Malaria case management according to the guidelines based on the *Integrated technical guidelines for frontline health workers*.^18^

^d^ Correctly asked or checked for the 7 IMCI danger signs and symptoms according to the *Integrated technical guidelines for frontline health workers*.^18^

^e^ Correct classification was defined according to the *Integrated technical guidelines for frontline health workers*.^18^

^f^ Correct “treatment/prescription” was defined according to the integrated technical guidelines.^18^

### Chief complaints

Of the 161 febrile children observed, most presented with symptoms and findings that were consistent with upper respiratory and gastrointestinal infections. The leading chief complaint was fever at 89.4% (144 children) followed by cough at 49.1% (79 children), diarrhoea at 24.8% (40 children), vomiting at 12.4% (20 children) and rash at 9.9% (16 children). Exit interviews revealed that non-pneumonia respiratory infections accounted for the highest proportion of diagnoses with 43% (69 children), followed by diarrhoea with 17% (27 children) and malaria with 10% (16 children).

### Skills assessments

The health workers assessed 82.6% of the surveyed children (133) for at least one danger sign, 47.2% (76) for two or more danger signs and 8.1% (13) for all four danger signs (Table 3). None of the 161 children were assessed for all seven signs and symptoms of febrile illness, and only 14.3% (23) were assessed for at least four. While health workers measured temperature in 94% of the children (147 of the total 156 children where temperature was checked), none of the health workers took a medical history and none completed all physical examination tasks as prescribed. Only 14.3% (23 health workers) did more than half of the required fever management tasks.

**Table 3:** Health-care workers’ assessment of 161 children with febrile illness, by type of health worker, Southern Province, Zambia, 2013

Assessment	No. (%)			
	Physician, clinical officer (*n* = 61)	Nurses (*n* = 78)	Other cadres^a^ (*n* = 22)	All encounters (*n* = 161)
**Assessment of danger signs**				
Able to drink or breastfeed	32 (53.3)	42 (53.8)	12 (57.1)	86 (54.1)
Vomits consistently	28 (47.5)	39 (50.6)	9 (42.9)	76 (48.4)
Had convulsions or is convulsing now	18 (31.0)	24 (30.8)	8 (38.1)	50 (31.8)
Unconscious or lethargic	27 (45.8)	21 (26.9)	2 (9.5)	50 (31.6)
Health worker asks or checks for at least one of the danger signs	54 (88.5)	65 (83.3)	14 (63.6)	133 (82.6)
Health worker asks or checks for at least two of the danger signs	31 (50.8)	36 (46.2)	9 (40.9)	76 (47.2)
Health worker asks or checks for all four of the danger signs	4 (6.6)	7 (9.0)	2 (9.1)	13 (8.1)
**Assessment of focal symptoms of febrile illness**				
Assess for cough or difficulty breathing	49 (83.1)	66 (84.6)	19 (90.5)	134 (84.8)
Assess for ear symptoms	21 (34.4)	15 (19.2)	10 (47.6)	46 (28.8)
Assess for neck stiffness	3 (5.1)	3 (3.9)	0 (0.0)	6 (3.8)
Assess for pallor	27 (45.0)	21 (27.3)	5 (23.8)	53 (33.5)
Assess all	0 (0.0)	0 (0.0)	0 (0.0)	0 (0.0)
**Overall appropriate assessment of febrile illness**				
Appropriate assessment of febrile illness^b^	0 (0.0)	0 (0.0)	0 (0.0)	0 (0.0)
Better than average assessment of febrile illness^c^	11 (18.0)	12 (15.4)	0 (0.0)	23 (14.3)

^a^ Includes 4 cleaning and maintenance staff, 1 community health assistant, 1 community health worker and 1 data clerk.

^b^ Health worker carried out all physical examination and history taking tasks according to the *Integrated technical guidelines for frontline health workers*.^18^

^c^ Health worker carried out more than half of the specified physical examination and history taking tasks according to guidelines.^18^

Note: Represented are the percentages of those observed to perform certain fever assessment skills, for each health-worker cadre, respectively. The total column denominators refer to the number of cases seen by each health-care worker cadre, summing up to clinical encounters with a line total of 161 children included in the study.

### Diagnosis and treatment

Table 4 and Table 5 present the health-care workers’ diagnosis and treatment of pneumonia and malaria respectively, as reported in exit interviews with caregivers. Only 27.8% (22 of the 79 children with diagnosis of cough or difficulty breathing) had their respiratory rate counted and 47.4% (9 of the 19 children with diagnosis of pneumonia) received appropriate antibiotic treatment. In contrast, over prescription of drugs was common. We found 45.8% (11 of the 19 children with a diagnosis of pneumonia) were inappropriately treated with amoxicillin, while 12.9% (11 of the 85 children with negative malaria rapid diagnostic test or microscopy results) were inaccurately classified as having malaria.

**Table 4:** Health-care workers’ diagnosis and treatment of pneumonia as reported in exit interviews with caregivers, Southern Province, Zambia, 2013

Evaluation characteristic	Total no. of interviews	No. of children (%)
**Diagnosis**		
Cough/difficulty breathing with respiratory rate counted	79	22 (27.8)
Fast breathing present in those checked	43	19 (44.2)
Appropriate pneumonia diagnosis^a^	19	4 (21.1)
**Treatment**		
Appropriate treatment^b^	19	9 (47.4)
Inappropriate treatment^c^	19	11 (45.8)

^a^ Children having tachypnea.

^b^ Fast breathing, treated with amoxicillin.

^C^ Not fast breathing, treated with amoxicillin.

Note: Exit interviews with caregivers were used to collect information on demographic characteristics of febrile children, chief complaint for the visit and counselling and drug dispensing practices of health workers attending to children. Children’s clinic card was reviewed to get information on care received.

**Table 5:** Health-care workers’ diagnosis and treatment of malaria as reported in exit interviews with caregivers, Southern Province, Zambia, 2013

Evaluation characteristic	Total no. of interviews	No. of children (%)
**Diagnostic testing**		
Tested only with RDT, where available^a^	153	84 (54.9)
Positive RDT result	84	7 (8.3)
Tested with RDT or microscopy	161	92 (57.1)
Positive RDT or microscopy test result	92	7 (7.6)
**Classification accuracy**		
Accurate classification^b^	7	7 (100)
Inaccurate classification^c^	85	11 (12.9)
**Diagnosis accuracy**		
Accurate malaria diagnosis^d^	18	7 (38.9)
Accurate non-malaria diagnosis^e^	154	74 (48.1)
**Malaria treatment**		
Classified as malaria and treated with ACT	18	8 (44.4)
Appropriate malaria treatment^f^	7	7 (100)
Appropriate non-malaria treatment^g^	154	153 (99.4)

ACT: artemisinin-based combination therapy; RDT: malaria rapid diagnostic test.

^a^ Ninety-two per cent of all facilities have  malaria rapid diagnostic test capability.

^b^ Positive RDT or microscopy results classified as malaria.

^c^ Negative RDT or microscopy results classified as malaria.

^d^ Children who actually had malaria diagnosed as having malaria.

^e^ Patients who don’t have malaria among all who tested negative for malaria.

^f^ ACT given where RDT or microscopy is positive.

^g^ No ACT when RDT or microscopy is negative.

Note: Exit interviews with caregivers were used to collect information on demographic characteristics of febrile children, chief complaint for the visit and counselling and drug dispensing practices of health workers attending to children. Children’s clinic card was reviewed to get information on care received.

Of all febrile children 57.1% (92) were tested for malaria using rapid diagnostic test or microscopy of which 8.3% (7) tested positive for malaria. All seven children appropriately received artemisinin-based combination therapy. Of 26 (13%) children who had negative rapid diagnostic test results and who received amoxicillin, only one received amoxicillin appropriately.

## Discussion

This study assessed the quality of clinical management of childhood fever at lower levels of the health system (health centres and second level hospitals), which typically manage the majority of childhood febrile disease burden. Upper respiratory tract infections accounted for almost half of febrile children visits and only 10% of children were diagnosed with malaria. While this was not an etiology study, clinical diagnoses were similar to those found in recent childhood fever etiology studies conducted in Africa. For example, in a large Tanzanian study, most (62%; 625 children who presented to outpatient care for fever) were diagnosed with acute respiratory infections, while only 11% (105 children) were diagnosed with malaria.^20^

In our study, we observed some deviations from practice as recommended by national IMCI guidelines for management of childhood febrile illness. These include inadequate assessment for danger signs, such as limited evaluations to determine fever etiology, over- and under-diagnosis of malaria and inadequate pneumonia management. The health workers evaluated danger signs related to poor oral intake in about half of all children with fever, assessed convulsions or lethargy in about a third, and counted respiratory rate in 28% of cases of suspected pneumonia. These findings contrast with one study conducted in rural Ghana, where the respiratory rate was counted in 4% of children presenting with cough or difficult breathing.^21^ Upper respiratory tract infections are highly prevalent in Africa and are predominantly non-pneumonia infections that are likely to be of viral etiology.^20^ In our study, the majority of health workers did not appropriately evaluate and classify children with pneumonia and upper respiratory tract infections, which highlights an important case management challenge. In a recent study of patients with febrile illness presenting to primary health-care facilities in Zambia, we found high proportions of these patients who received overtreatment with antibiotics (61% and 75%, respectively) for those diagnosed with diarrhoea and upper respiratory tract infections,^12^ indicating a need to improve diagnostic capacity for non-malaria causes of febrile illness and limit antibiotic use to patients with definite bacterial infections. As malaria prevalence declines, high-quality care for febrile children requires consistent use of malaria diagnostic tests and formulation of a differential diagnosis based on symptoms and physical signs.^22^^,^^23^

Despite increasing availability of malaria rapid diagnostic tests in Zambia^8^ and health-care worker training in their use, little over half of febrile children in this study were tested for malaria with either rapid diagnostic test or microscopy. Of those tested, 13% of children with a negative test result were still classified as having malaria. Managing non-malaria fever continues to pose a challenge globally.^24^ WHO estimates that in the WHO African Region, over 60% of children with fever receive a blood test (rapid diagnostic test or microscopy) at public facilities.^25^ In a recent nationwide study in Zambia on fever management, among over 700 patients where half of the children were younger than 5 years, 75% were tested for malaria, and testing was associated with reduced antibiotic prescribing.^12^ In a Ghanaian study on childhood fever, where malaria rapid diagnostic tests were fully available, less than half of febrile children were given a malaria test. In the same study, 45% of artemisinin-based combination therapy prescriptions conformed to guidelines and were appropriately used to treat malaria.^26^

In our study, all patients who tested positive using microscopy or a rapid diagnostic test received artemisinin-based combination therapy, in contrast to only one with a negative test. This indicates a higher level of compliance with rapid diagnostic testing results than previously observed in a study in Zambia, where 36% of those with a negative rapid diagnostic test result were prescribed an antimalarial.^17^ Adherence to rapid diagnostic test results has varied widely in studies conducted in Africa.^27^ While adherence to positive results tends to be appropriate, compliance with negative results still needs to improve.^28^

Withholding antimalarial drugs from patients with a negative rapid diagnostic test result seems a safe approach, even for children living in areas highly endemic for malaria.^1^ While the use of malaria rapid diagnostic testing has reduced the consumption of antimalarial drugs, in some settings, it has increased the use of antibiotics,^29^ replacing the problem of misuse of antimalarial drugs with antibiotic overtreatment.^12^ The clinical algorithm for IMCI is designed to reduce unnecessary antibiotic treatment, however, in most cases, not adequately following the IMCI guideline still leads to some overtreatment because of the poor specificity of respiratory rate to diagnose pneumonia in patients with cough or difficult breathing.^30^ While the clinical overlap of pneumonia with malaria makes their management at peripheral facilities challenging, evidence exists that with effective training, provision of job aids, and regular supportive supervision, community health workers are able to correctly classify malaria and pneumonia and provide appropriate treatment.^31^

In our study, less than half of children diagnosed with pneumonia received the appropriate amoxicillin treatment, yet it was also often prescribed in children without pneumonia. According to WHO guidelines, about 80% of children with respiratory symptoms do not need antibiotics and can receive supportive care for cough and cold.^24^ A hospital trial of 900 children in Pakistan showed that even severe pneumonia can be managed safely at community level without antibiotics.^32^ Several studies in Pakistan have shown that community health workers can recognize chest indrawing and treat children in the community.^33^^,^^34^ Training community health workers in the management of severe pneumonia can reduce treatment failure by half and can lead to substantial household savings. A study in Pakistan showed that costs were 1.40 United States dollars (US$) for cases managed by a health worker versus US$ 7.60 for treatment costs without.^35^ As a result of these and other studies, current IMCI guidelines recommend that pneumonia with chest indrawing but no other danger signs be managed at the peripheral level.^24^

Our study further documented that few providers had the knowledge and skills to identify danger signs; make the right diagnosis; and initiate appropriate treatment following national guidelines. The results illustrate the need to enhance the availability and awareness of current diagnosis and treatment guidelines that providers will be able to use. District and facility managers can support health-care workers by providing structural support, through systematic coaching and supportive supervision, to recognize signs, symptoms and pertinent history to correctly categorize and treat febrile illness, such as measuring respiratory rate. Access to guidelines and job aids as well as structural and organizational changes such as providing triage facilities dedicated to managing children in larger facilities are ways to provide that support.

Our study has limitations. First, the relatively small numbers of health facilities and health workers included in the study limits the generalizability of our results beyond Southern Province. However, our findings are consistent with challenges related to clinical assessment and management of children with fever reported elsewhere in Zambia^12^ and other African countries.^20^ Second, the assessment of appropriateness of health worker assessments relied on maternal recall of symptoms and we did not independently verify disease etiologies in our study. The total number of children presenting with fever recorded in our study was less than anticipated. Finally, our observations of health workers were subject to the reactive effects of study arrangements, potentially modifying their behaviours when aware of being observed. However, the Hawthorne effect – i.e. health-care providers changing their behaviour when being observed – has been found to have limited influence on health workers’ practices during consultations with ill children in similar health-care settings in Benin.^36^

In conclusion, this study revealed various health systems challenges that may have contributed to a substantial proportion of children not receiving early and appropriate treatment for febrile illness. To improve management of febrile illness in Zambia, interventions need to focus on improving identification of severe illness and strengthening diagnostic capacity (both clinical and laboratory or point-of-care tests). In addition, health workers needs to improve prescribing practices for antibiotic therapy and to enhance the supply chain for better access to IMCI commodities, such as malaria rapid diagnostic tests, antimalarials and antibiotics. Health workers in Zambia need improved guidance and training to appropriately use and respond to malaria test results and to effectively manage childhood febrile illness.

Tools for the management of childhood fevers at peripheral health facilities are available in the form of international and national guidelines. The Zambian health system needs innovative approaches to improve their application in the management of childhood febrile illness. Improvement will ensure appropriate treatment and referral of patients with non-malaria febrile illness, especially for respiratory tract infections. It will also minimize unnecessary use of antimalarial drugs, thus reducing morbidity, mortality and related health-care costs.
